# Primary gallbladder neuroendocrine neoplasm: A case report of grade 1 well-differentiated neuroendocrine tumor

**DOI:** 10.1016/j.ijscr.2024.110604

**Published:** 2024-11-15

**Authors:** Ardenne Ko, Morgan MacKenzie, Kenrry Chiu, Wan Wan Yap, George Melich, Shawn MacKenzie

**Affiliations:** a Department of Surgery, Royal Columbian Hospital, Fraser Health Authority, 330 East Columbia Street, New Westminster, British Columbia V3L 3W7, Canada; b University of British Columbia, Faculty of Medicine, Department of Surgery, 2775 Laurel Street, 11th Floor, Vancouver, British Columbia V5Z 1M9, Canada; c Department of Pathology, Royal Columbian Hospital, Fraser Health Authority, 330 East Columbia Street, New Westminster, British Columbia V3L 3W7, Canada; d Department of Radiology, Surrey Memorial Hospital, Fraser Health Authority, 13750 96th Avenue, Surrey, British Columbia V3V 1Z2, Canada

**Keywords:** Case report, Gallbladder tumor, Gallbladder neuroendocrine neoplasm, Neuroendocrine tumor, Cholecystectomy

## Abstract

**Introduction:**

Neuroendocrine neoplasm (NENs) make up approximately 2–3 % of gallbladder malignancies, while only 0.5 % of all NENs develop in the gallbladder. Most Gallbladder neuroendocrine neoplasms (GB-NENs) are discovered incidentally during pathological examinations post-cholecystectomy.

**Case presentation:**

70-year-old male presents with an incidentally discovered 2.2 cm enhancing intraluminal soft tissue mass on abdominal CT scan. The mass demonstrates restricted diffusion on MR imaging, concerning for gallbladder malignancy. Radical cholecystectomy, confirms primary gallbladder neuroendocrine tumor (GB-NET). No adjuvant therapy was recommended at multidisciplinary cancer conference review. The patient is currently disease free at 18 months follow up.

**Discussion:**

The management of GB-NEN remains challenging, due to the lack of specific clinical manifestations and typical imaging features preoperatively. GB-NENs are usually asymptomatic, and the paucity of reported imaging characteristics makes prospective diagnosis of GB-NENs challenging. GB-NEN tend to be larger in size, demonstrating well defined, intact mucosa, with a thick rim of hyperintensity on diffusion weighted images (DWI). Distinguishing between gallbladder neuroendocrine carcinoma (GB-NEC) and gallbladder neuroendocrine tumor (GB-NET) on pathologic evaluation is essential in developing a treatment plan. GB-NETs have superior survival compared to GB-NECs. GB-NETs can be managed utilizing a cholecystectomy with portal lymphadenectomy +/− segment 4b/5 liver resection.

**Conclusion:**

GB-NETs may achieve curative resection, if identified at an early disease stage.

## Highlights

• Primary gallbladder neuroendocrine neoplasms are a rare type of gallbladder cancer.
• Primary gallbladder neuroendocrine neoplasms are difficult to diagnose preoperatively due to the absence of symptoms and nonspecific radiological findings.
• Radical cholecystectomy remains the treatment of choice to confirm pathologic diagnosis of primary gallbladder neuroendocrine tumor, and to attempt curative resection.

## 1 Introduction

Primary gallbladder neuroendocrine neoplasm (GB-NEN) is a rare type of tumor that stems from neuroendocrine cells, found throughout the body. Neuroendocrine neoplasms (NENs) exhibit a spectrum of behavior, ranging from indolent behavior to aggressive malignancy with metastatic disease. NENs make up approximately 2–3 % of gallbladder malignancies, while only 0.5 % of all neuroendocrine tumors develop in the gallbladder [1]. NENs are more commonly found in the gastrointestinal tract. Functional NENs produce hormones that produce non-specific symptoms including weight loss, discomfort, and abdominal pain [2]. GB-NENs may have a poor prognosis as the diagnosis is rarely made preoperatively due to vague symptomatology, and lack of specific imaging features. Most GB-NENs are discovered incidentally during pathological examinations post-cholecystectomy. Surgical resection remains the recommended treatment to confirm the diagnosis and potentially cure GB-NENs [3,4]. The case is reported following SCARE guidelines [5].

## 2 Presentation of case

A 70-year-old male, 2 months post-operative from a right nephrectomy, presents with sudden epigastric pain, light-headedness, and nausea. An emergency department abdominal computed tomography (CT) scan identified duodenal perforation, incidentally, revealing an enhancing soft tissue mass within the gallbladder. The patient underwent an urgent laparoscopic modified Graham-patch duodenal ulcer repair.

### 2.1 Past medical history

The patient is a previous smoker, with chronic obstructive pulmonary disease (COPD) and remote COVID-19. Comorbidities include acid reflux with Barrett's esophagitis, remote bilateral inguinal hernia repair, and atrial fibrillation. Two months prior to presenting in the emergency room, the patient underwent a right nephrectomy for a 6 cm renal cell carcinoma, clear cell type, Fuhrman nuclear grade 3/4 with no lymphovascular invasion. Immunohistochemical stains were positive for PAX8, AE1/AE3, E-cadherin, CD10, and vimentin; the tumor was negative for CK7, CD117, racemase, ALK, and HMB45; the tumor showed focal, scattered staining for Melan-A and very weak cytoplasmic staining for CAIX. TFE3 FISH testing on the tumor was negative.

### 2.2 Investigations

**Fig. 1:**
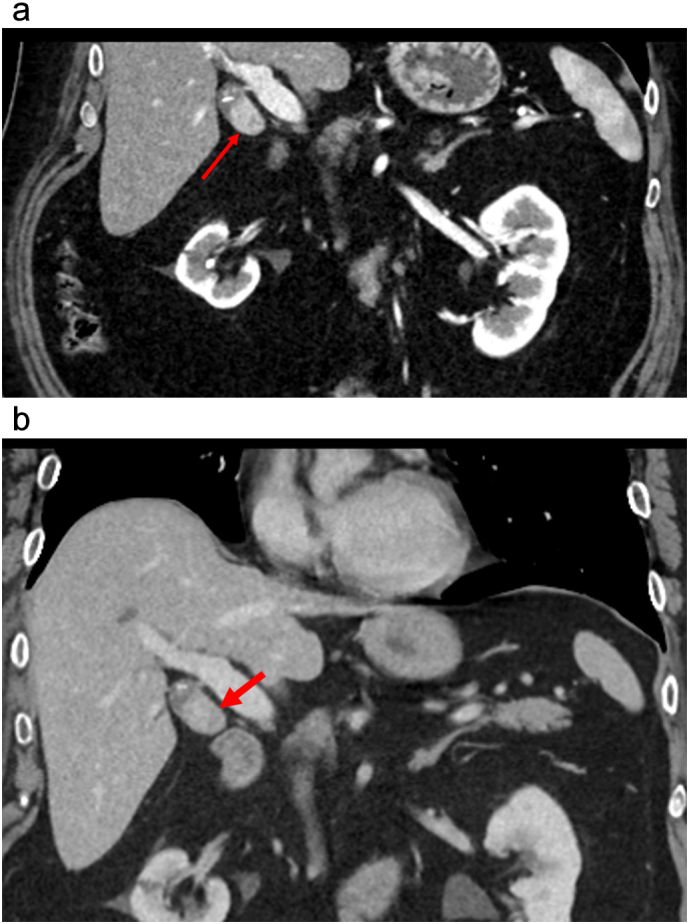
A. Coronal CT arterial phase showing arterial phase enhancing lesion (arrow). B. Coronal CT portal venous phase enhancing lesion (arrow).

**Fig. 2:**
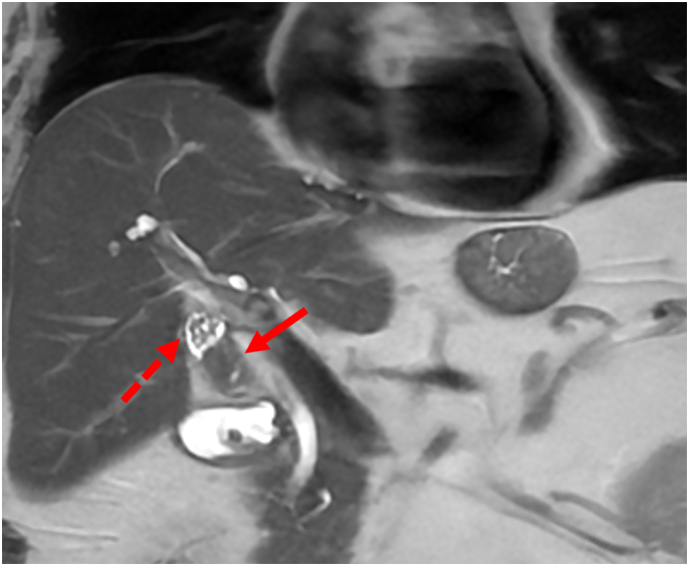
Coronal T2W sequence. (Solid arrow shows the mass and dashed arrow – gallstones).

**Fig. 3:**
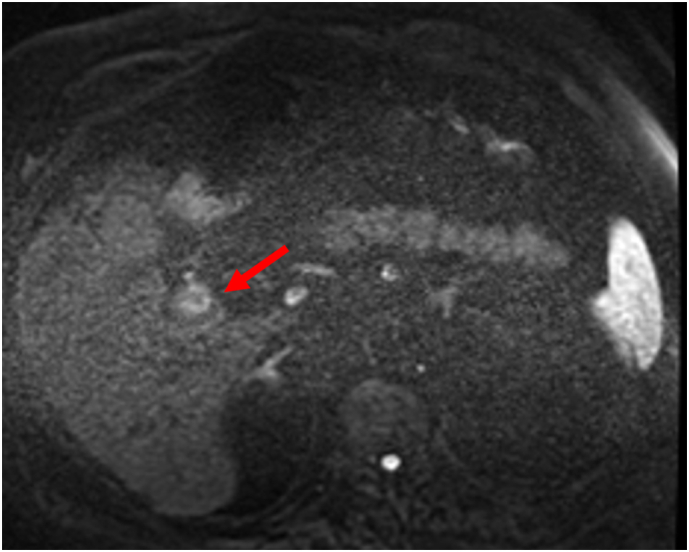
Ax DWI b-1000 shows rim high signal at periphery (arrow).

The emergency department CT scan incidentally revealed a 2.2×2.0 cm enhancing soft tissue mass centered in the gallbladder neck. The mass appeared to be intraluminal demonstrating arterial phase hyperenhancement, persistent enhancement on portal venous phase and washout on delayed phase imaging (Fig. 1A and B). Magnetic resonance Imaging (MRI) confirmed the presence of a 2.3 × 2.0 cm solid enhancing mass at the inferior margin of the gallbladder neck (Fig. 2). The mass demonstrates restricted diffusion (Fig. 3), no evidence of gallbladder wall thickening, and no evidence of metastatic liver or abdominal disease. Based on the imaging appearance, the differential diagnosis included primary gallbladder adenocarcinoma, adenomyomatosis or metastatic renal cell carcinoma.

### 2.3 Therapeutic intervention

Due to the concern for gallbladder malignancy, the patient underwent a laparoscopic converted to open cholecystectomy. At the time of operation, a 2 cm mass was located in Hartmann's pouch. A radical cholecystectomy with complete portal lymph node dissection was undertaken. Due to the position of the mass in the porta hepatis, a segment 4B/5 hepatic resection was not indicated. The patient was discharged home after surgery with no 90-day postoperative complications.

### 2.4 Pathology

**Fig. 4:**
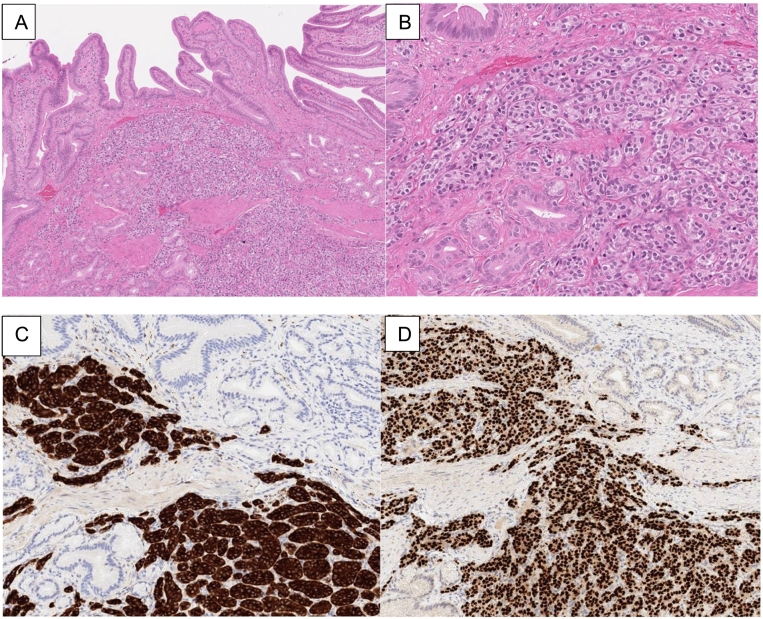
Well-differentiated neuroendocrine tumor of the gallbladder. The tumor consists of nests and trabeculae of monotonous cells and is centered in the wall of the gallbladder wall (A, B). The tumor is diffusely positive for synaptophysin (C) and PAX8 (D) by immunohistochemistry.

Gross examination of the specimen revealed a 2.0 cm ill-defined solid pale-yellow tumor in the gallbladder wall near the cystic duct. No calculus was identified. Microscopic examination of the tumor demonstrated nests and trabeculae of monotonous epithelioid cells with pale cytoplasm and stippled chromatin (Fig. 4A,B). The tumor mitotic rate was <2 per 2 mm^2^. The tumor involved the muscular wall of the gallbladder, with extension into the overlying mucosa and perimuscular connective tissue. The background gallbladder mucosa showed intestinal metaplasia but no epithelial dysplasia or carcinoma. Immunohistochemistry stains showed the tumor was positive for synaptophysin (Fig. 4C), chromogranin, INSM1, AE1/AE3, PAX8 (MRQ-50 clone) (Fig. 4D), CDX2 (patchy, weak), MOC31, and CD10 and was negative for claudin-4, CK7, CK20, GATA3, TTF1, p40, and S100; Ki-67 index was 1.5 %. The gallbladder tumor was morphologically distinct from the previously resected renal cell carcinoma, with additional renal cell tumor immunohistochemistry staining negative for synaptophysin, chromogranin and INSM1.

### 2.5 Post-operative course

The gallbladder tumor was consistent with a grade 1 well-differentiated neuroendocrine tumor (NET). Due to the rarity of primary NETs of the gallbladder, a clinical evaluation for an alternative primary site (pancreatic or gastrointestinal NET) was undertaken. A staging 68Ga-DOTATOC PET/CT post radical cholecystectomy demonstrated no evidence of an alternative primary neuroendocrine tumor. Perioperative Chromogranin A was elevated at 1087 (reference range < 94 μg/L) and remains elevated (864 μg/L at 1 year). The elevation may be explained by proton pump inhibitor use. Multidisciplinary cancer conference recommended no systemic therapy, advising somatostatin analog treatment if recurrence of the gallbladder neuroendocrine tumor (GB-NET) is identified. The patient remains disease free at 18 months post-radical cholecystectomy, without evidence of disease recurrence, and/or metastatic spread on surveillance abdominal CT imaging.

### 2.6 Patient perspective

The surgical team delivered the news of a rare form of gallbladder cancer, discussing the prognosis and treatment. When I was told that I had a tumor on my gallbladder, I was very surprised. I was concerned that my kidney cancer may have spread, and worried for my health down-the-road. I thought “ok, let's take it out.” Today, I feel fine and do not have any symptoms of any sort.

## 3 Discussion

NENs are most commonly found in the gastrointestinal tract (66 %) [6], with GB-NENs making up only 0.5 % of all NETs [1]. Primary GB-NENs rarely develop in the gallbladder due to the absence of neuroendocrine cells in the gallbladder wall, leading to speculation that gallbladder neuroendocrine tumors may develop from intestinal metaplasia from the gallbladder epithelium, undifferentiated multipotent gallbladder stem cells [7], and/or within the neck of the gallbladder which may harbour neuroendocrine cells [6]. The WHO classifies neuroendocrine neoplasms into three categories: neuroendocrine tumor (NET), neuroendocrine carcinomas (NECs), and mixed neuroendocrine-non-neuroendocrine neoplasms [8].

The incidence of GB-NEN, and more specifically GB-NET, remains difficult to determine due to the relatively low number of reported cases within the world literature. In addition, the term “gallbladder neuroendocrine tumor” is often used universally to describe neuroendocrine neoplasms, rather than well-differentiated neuroendocrine tumors. Cen et al. performed a SEER database review in 2019, which identified 248 cases of primary GB-NENs over a 11-year-period, with 70 cases (28 %) reported as “carcinoid tumor” or GB-NET [9]. Over a similar period, Ayabe et al. reviewed the NCDB database identifying 2.3 % of gallbladder tumors in the database as NEN (754/32,457 patients) [1]. A recent single institution study at Peking Union Medical Hospital identified 22 NENs in 11,260 patients with gallbladder tumors between 2012 and 2021 [10]. 18 % of NENs (4 patients) were classified as NETs (Ki-67 % < 1 %) revealing an incidence of 0.035 % in this study [10].

Management of GB-NEN remains challenging, due to difficulty in diagnosing NEN preoperatively since there is a lack of specific clinical manifestations and typical imaging features. GB-NENs are usually asymptomatic and thus are more difficult to diagnose compared to digestive system NENs [10]. The paucity of reported imaging characteristics makes prospective diagnosis of GB-NENs challenging. Bae et al. compared 21 GB-NEN and 42 cases of GB adenocarcinoma, identifying several classic MR-Imaging features [11]. GB-NEN tends to be larger in size, demonstrating well defined, intact mucosa, with a thick rim of hyperintensity on diffusion weighted images (DWI). Due to the asymptomatic nature of GB-NEN, presentation with liver metastases is common. While these imaging features have been identified in GB-NEN, there is significant cross-over with gallbladder adenocarcinoma [12]. As gallbladder adenocarcinoma increases in size, the tumor tends to become more infiltrative than GB-NENs. Our patient's tumor was identified incidentally on CT scan and was small in size at the time of presentation. The tumor was well defined, without breach of the mucosa, and free of metastases. Dynamic contrast imaging revealed arterial phase hyperenhancement, typical of NENs. The GB-NET mass demonstrated rim hyperintensity on DWI, despite the relatively small size of the lesion and poor resolution of the images obtained due to motion artefacts.

Database analyses suggest that patients with GB-NEN demonstrate better overall survival with surgical resection, while prognosis appears to be reliant on tumor grade [1,4,9]. Distinguishing between GB-NEC and GB-NET is essential in developing a treatment plan. NEC demonstrate a poor overall survival (median survival 9.8 months [3)], when compared to NET. Gogna et al. reviewed of 482 GB-NEN patients in the SEER database from 1973 to 2016, identifying 10 % of GB-NEN as grade 1 GB-NET, which demonstrated a median survival of 150 months [4]. Due to the difficulty in preoperatively distinguishing NETs from NECs, the initial treatment for gallbladder neuroendocrine neoplasms remains radical cholecystectomy [2], for pathologic confirmation and guiding subsequent management. Patients who underwent radical cholecystectomy demonstrated superior overall survival when compared to unresectable lesions (median survival 111.0 months vs. 8.3 months) [4].

Our patient is a 70-year-old male, diagnosed with incidental abdominal CT scan imaging due to unrelated abdominal pain, with pathological confirmation of GB-NET after surgical resection. The patient underwent a radical cholecystectomy, received no adjuvant systemic therapy, and is currently disease free at 18 months.

## 4 Conclusion

Reported is a case of GB-NET which adds to the limited knowledge on GB-NEN, increasing the database of radiological images and confirming cholecystectomy leads to long-term disease-free survival.

## Consent

Written informed consent was obtained from the patient for publication of this case report and accompanying images. A copy of the written consent is available for review by the Editor-in-Chief of this journal on request.

## Ethical approval

Fraser Health Research Ethics Board approval for ethics exemption for medical case reports.

## Guarantor

Shawn MacKenzie.

## Research registration number

N/A.

## Funding

Authors received no sources of funding.

## Author contribution

Ardenne Ko – manuscript preparation, manuscript concept development, literature review.

Morgan MacKenzie - manuscript preparation, manuscript concept development, literature review.

Kenrry Chiu - manuscript preparation, pathology interpretation, manuscript editing.

Wan Wan Yap - manuscript preparation, radiology interpretation, manuscript editing.

George Melich – manuscript editing.

Shawn MacKenzie - manuscript preparation, case report/surgical care interpretation, manuscript editing, manuscript concept development, literature review.

## Conflict of interest statement

The authors have no conflict of interest to disclose.
